# Characterization of H_3_PO_4_-Treated Rice Husk Adsorbent and Adsorption of Copper(II) from Aqueous Solution

**DOI:** 10.1155/2014/496878

**Published:** 2014-02-11

**Authors:** Ying Zhang, Ru Zheng, Jiaying Zhao, Fang Ma, Yingchao Zhang, Qingjuan Meng

**Affiliations:** ^1^School of Resource and Environment, Northeast Agricultural University, Harbin 150030, China; ^2^State Key Laboratory of Urban Water Resource and Environment, Harbin Institute of Technology, Harbin 150090, China

## Abstract

Rice husk, a surplus agricultural byproduct, was applied to the sorption of copper from aqueous solutions. Chemical modifications by treating rice husk with H_3_PO_4_ increased the sorption ability of rice husk for Cu(II). This work investigated the sorption characteristics for Cu(II) and examined the optimum conditions of the sorption processes. The elemental compositions of native rice husk and H_3_PO_4_-treated rice husk were determined by X-ray fluorescence (XRF) analysis. The scanning electron microscopic (SEM) analysis was carried out for structural and morphological characteristics of H_3_PO_4_-treated rice husk. The surface functional groups (i.e., carbonyl, carboxyl, and hydroxyl) of adsorbent were examined by Fourier Transform Infrared Technique (FT-IR) and contributed to the adsorption for Cu(II). Adsorption isotherm experiments were carried out at room temperature and the data obtained from batch studies fitted well with the Langmuir and Freundlich models with *R* ^2^ of 0.999 and 0.9303, respectively. The maximum sorption amount was 17.0358 mg/g at a dosage of 2 g/L after 180 min. The results showed that optimum pH was attained at pH 4.0. The equilibrium data was well represented by the pseudo-second-order kinetics. The percentage removal for Cu(II) approached equilibrium at 180 min with 88.9% removal.

## 1. Introduction

Currently, the removal of heavy metal contaminants from aqueous wastewater is one of the most important environmental issues. Even though this issue has been studied for several years, effective and precise treatment methods are still scanty [1]. Excessive toxic heavy metals are often discharged by a number of industries, such as metal plating facilities, mining operations, battery manufacturing, and tanneries [2, 3]. Heavy metals are not biodegradable and can lead to accumulation in living organisms, causing various diseases and disorders [4]. Monitoring and subsequent removal of toxic heavy metals from industrial wastewater have been made mandatory before their discharge into the environment [5]. Copper, one of the heavy metals, is a widely used industrial metal for machinery and metallurgy, plumbing, electrical wiring, and transportation industry. It is also used for anticorrosion and as a decorative coating on metal alloys [6]. Copper ion is found in wastewaters of industries such as metal cleaning, electroplating industry, refineries, and wood preservatives, paper and pulp. Copper also is an essential microelement of the body. However, excessive intake of Cu(II) by human beings leads to gastrointestinal irritation, hepatic and renal damage, cirrhosis, and central nervous problems [7]. The Cu(II) is toxic to lower organisms and farm crops as well. In addition, the Cu(II) has great toxicity to crops and particularly is considered the most toxic heavy metals regarding to their degree of toxicity to the crops. The pollution of Cu(II) is mainly due to industrial effluents discharge of electroplating, hardware, petrochemical processing, and chemical industry [8]. The World Health Organization (WHO) recommended that the maximum acceptable concentration of Cu(II) in drinking water is 1.5 mg/L [9]. In China, the Ministry of Environment and Forests has set the national standards of 0.5 mg/L for Cu(II) for safe discharge. For this reason, the discharge of industrial effluents containing Cu(II) must be stringently regulated in order to reduce environmental pollution.

Conventional methods applied to remove toxic heavy metals from effluents include chemical precipitation [10], ion exchange [11], carbon adsorption [12], and membrane separation process [13]. At present, adsorption is widely accepted in environmental treatment applications throughout the world compared with the other methods [14]. Among all kinds of adsorption materials, activated carbon adsorption has been regarded as an efficient and major technology, but the process is expensive [15]. To all intents and purposes, some main limitations are existent in these alternatives, such as expensive cost, labor-intensive operation, and low efficiency [16]. Therefore, more approaches have been investigated for the development of low-cost adsorbents with a good sorption capacity to remove heavy metal ions from wastewater. In recent years, considerable attention has been devoted to the study of application of agricultural materials as adsorbents. Natural materials have the advantages of large quantities, low cost, and good sorption capacity. They are always the unutilized materials but they have high potential to be used as adsorbents for heavy metals removal [17]. Many agricultural and forest waste products have been studied for effluent treatment as an adsorbent, such as rice husk [18], peanut shells [19], corncobs [20], wheat bran [21], and sawdust [22]. In this sense, various solid wastes can be available for removing Cu(II) from aqueous effluents.

In China, rice husks have a large quantity of production generated as a byproduct of rice processing. Rice husk is a kind of fibrous material containing a huge amount of silicon with the content being approximately 96.34% [23]. In addition, cellulose, hemicellulose, and lignin are the main organic compounds of rice husk. This composition of rice husk makes it possible to be regarded as an adsorbent. Currently, people have taken more interest in the application and investigation of rice husk as an adsorbent. Previous researches reveal that rice husk has been used for removing ionic dyes from aqueous solutions [24] and pretreated rice husk has been used for the sorption of cadmium from effluents [25]. In our preliminary research, we studied the adsorption of heavy metals using untreated rice husk during in our preliminary research. The results obtained showed that rice husk can be considered unpublished results of our lab as good sorbent for some metal ions. Since pretreatment was found to improve sorption capacity of rice husk, we chose H_3_PO_4_-treated rice husk as the adsorbent, as it is a simple and low-cost chemical modification method.

The objective of this work was to examine the adsorption characteristics of H_3_PO_4_-treated rice husk to adsorb Cu(II) ions from an aqueous solution. Therefore, the adsorption isotherms and kinetics were investigated. The effects of sorption parameters such as pH and contact time were examined. In addition, the adsorption mechanism of Cu(II) was discussed in this research. Therefore, the potential of H_3_PO_4_-treated rice husk was evaluated for use in wastewater treatment.

## 2. Experimental

### 2.1. Materials and Methods

Fresh rice husk used in this work was obtained from a local farm in Harbin city. The impurities in rice husk were first picked out. Then the rice husk was washed thoroughly with distilled water before oven drying at 60°C until a constant weight was attained. The sorbent obtained (hereafter raw rice husk) was put into a cool and dry place. All reagents were of analytical grade and distilled water was used throughout. The Cu(II) stock solutions, at 1 g/L concentrations, were prepared in distilled water by dissolving accurate quantity of Cu(NO_3_)_2_ reagent.

The Cu(II) concentrations of supernatant sample were determined by using atomic absorption spectrometry (Model AA6800, Shimadzu, Japan). The X-ray fluorescence (XRF) spectrum analysis (Model Axios PW4400, PANalytical) was conducted for the element composition of raw rice husk and H_3_PO_4_-treated rice husk. Analysis of functional groups of H_3_PO_4_-treated rice husk and H_3_PO_4_-nontreated rice husk loaded with Cu(II) was obtained by Fourier Transform Infrared spectrophotometer (Nicolet Magne 750). The surface morphology of raw rice husk and H_3_PO_4_-treated rice husk was carried out by using a scanning electron microscope (Model S-3400N, HITACHI). The details are shown in Table 1.

### 2.2. Chemical Pretreatment on Rice Husk

In order to increase the specific surface of rice husk, the rice husk was ground using a disintegrator and after sieving the particle size *⩽*1 mm was retained for further experiment. This rice husk was mixed with 1 M of H_3_PO_4_ solution at room temperature for 24 h in 500 mL with a stirring speed of 150 rpm so that the reagents were fully adsorbed onto the raw material [26]. After this treatment, the modified rice husk was filtered and washed with distilled water for several times until the pH reached a constant value. Later on, this adsorbent was oven-dried at 110°C for 4 h. Then the H_3_PO_4_-treated rice husk was obtained.

### 2.3. Batch Adsorption Studies

The effect of pH was studied by varying the initial pH from 1 to 7 of 50 mL Cu(II) solutions at concentration of 5 mg/L and sorbent dose of 2 g/L by adjusting the pH with 0.1 M of NaOH and 0.1 M of HNO_3_. After shaking the flasks for 3 hours at room temperature (25°C) and 130 rpm, the mixture was filtered using an acid-cleaned 0.45 *μ*m Millipore filter and the concentration of Cu(II) in the filtrate was determined by atomic absorption spectrometry. The flasks were hermetically sealed to avoid volatilization of solutions in the oscillatory process. All results were obtained in duplicate tests and were consequently the averaged values of duplicate tests.

The kinetic experiments were performed in continuously stirred flask containing 50 mL Cu(II) solutions at concentration of 5 mg/L from 15 min to 300 min, at sorbent dose of 2 g/L and pH 4. Likewise, the mixture was shaken in a constant temperature oscillator at 25°C and 130 rpm. After filtering the mixture, the concentration of Cu(II) in the filtrate was determined by atomic absorption spectrometry.

Sorption isotherms were conducted at sorbent dose 2 g/L and the concentration of Cu(II) was varied from 5 mg/L to 50 mg/L in 150 mL conical flask containing 50 mL Cu(II) solutions. The pH was adjusted to 4 (the optimum pH value) and the mixture was shaken in a constant temperature oscillator at 130 rpm and temperature of 25°C for 3 hours.

The percentage removal of metal ions and equilibrium adsorption amount of Cu(II), *q*_*e*_ (mg/g), were calculated by using the following relationships:

 Percentage removal of metal ions = 100(*C*_0_ − *C*_*e*_)/*C*_0_,
 Adsorption amount of Cu(II) per gram (g) of
 adsorbent (mg/g), *q*_*e*_ = (*C*_0_ − *C*_*e*_)*V*/*w*,

where *C*_0_ is the initial concentration of Cu(II) (mg/L), *C*_*e*_ is the equilibrium concentration of Cu(II) (mg/L), *V* is the volume of the solution (L), and *w* is the mass of the adsorbent (g).

## 3. Results and Discussion

### 3.1. Characterization of H_3_PO_4_-Treated Rice Husk

**Figure 1:**
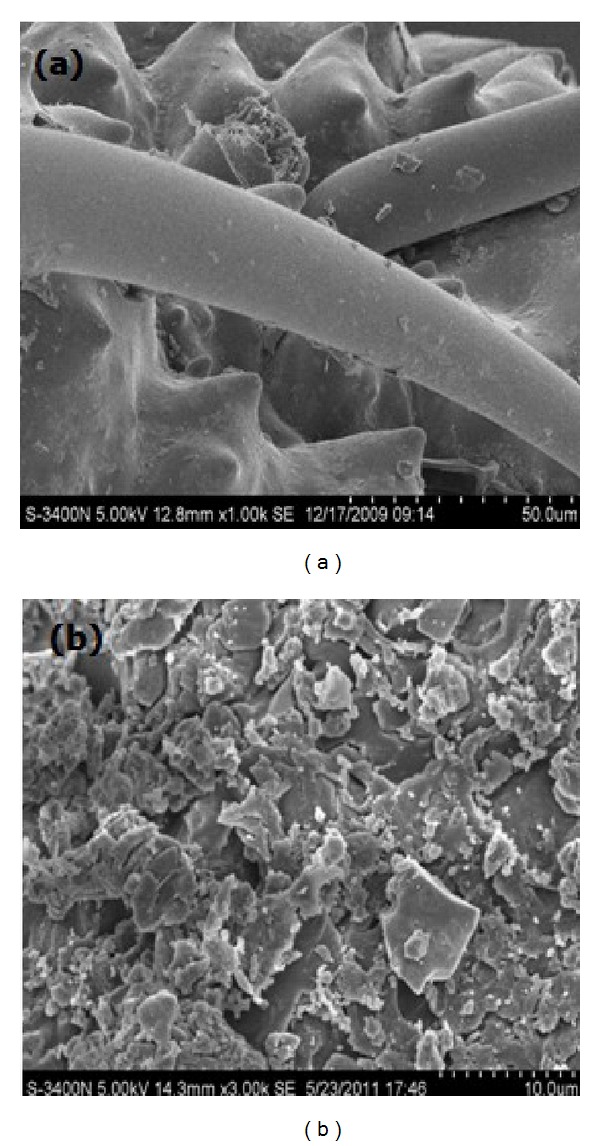
Scanning electron micrograph of (a) rice husk with 1000 times of magnification and (b) H_3_PO_4_-treated rice husk with 3000 times of magnification.

Rice husk consists of 32.24% of cellulose, 21.34% of hemicellulose, 21.44% of lignin, and 15.05% of mineral ash as well as high percentage of silica in its mineral ash, which is approximately 96.34% [23]. Scanning electron micrographs of native rice husk and H_3_PO_4_-treated rice husk are shown in Figure 1.

At low-power electron microscope, some regular and criss-cross framework construction can be seen on the surface of the native rice husk in Figure 1(a). And there were many embossments approximated cones and epidermal villi-like needles between the two lines of framework. This structure made rice husk be a good adsorbent. And after being modified, the material was more beneficial to the adsorption of metal ions. The irregular surface and porous structure are observed in Figure 1(b). The dark spots represent some pore openings and cavities which may facilitate the solution flow into the pore and enhance the adsorption kinetics. This morphological structure of the material is conductive to the uptake of metal ions. In addition, based on the fact that high amounts of silica are concentrated on the external epidermis of rice husk, it can be concluded that H_3_PO_4_-treated rice husk presents an adequate morphological profile for the adsorption of metal ions. The elemental compositions of native rice husk and H_3_PO_4_-treated rice husk using X-ray fluorescence (XRF) spectra analysis are shown in Table 2.

The different elements have the characteristic X-ray peak of different wavelengths. And the fluorescence intensity of each spectral line is related to the element concentration. The XRF spectrometer was used to analyze the elemental composition of adsorbents. Results showed that H_3_PO_4_-treated rice husk has an increase in the relative quantities of sodium (Na), silicon (Si), and phosphorus (P) and a decrease in potassium (K) and calcium (Ca) compared to raw rice husk. These variations of element content may somewhat influence the Cu(II) adsorption onto the adsorbents by ion exchange. Increase in Cu(II) uptake by H_3_PO_4_-treated rice husk is probably due to the higher concentrations of more easily exchangeable Na and Si.

**Figure 2:**
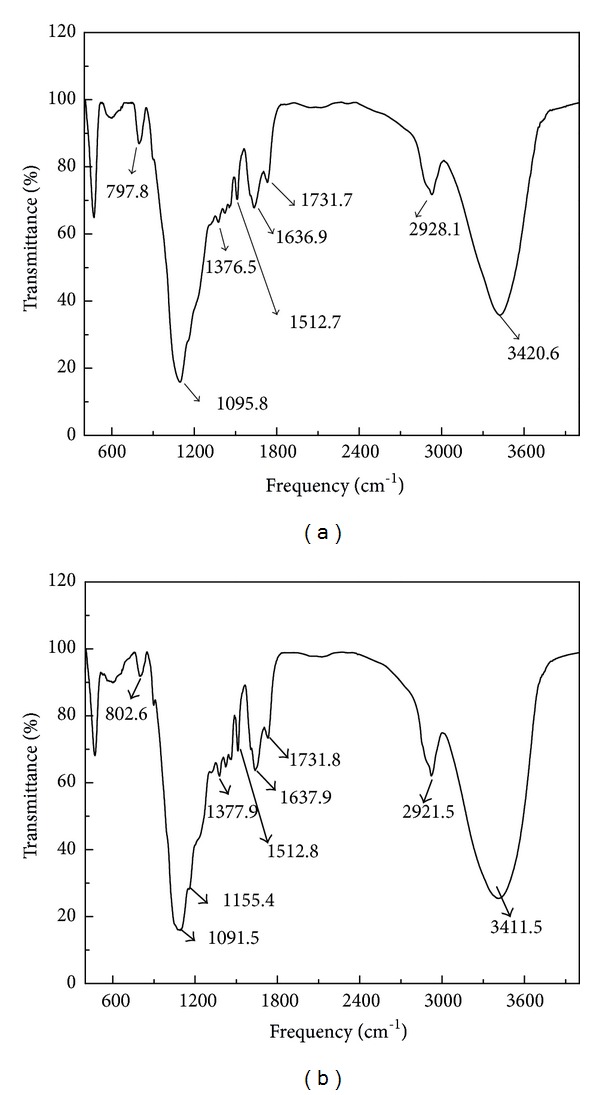
Fourier Transform Infrared (FT-IR) spectrum of (a) H_3_PO_4_-treated rice husk and (b) H_3_PO_4_-treated rice husk loaded with Cu(II).

The FT-IR technique, as an important tool to identify the characteristic of functional groups, can be used to understand the chemical compositions of the adsorbents material, which are very important for the uptake of metal ions. The FT-IR spectra of the blank and H_3_PO_4_-treated rice husk loaded with Cu(II) are shown in Figure 2.

Some fundamental frequencies of H_3_PO_4_-treated rice husk and respective possible band frequencies can be seen in the FT-IR spectrum. The wave number range of FT-IR spectrum is from 400 cm^−1^ to 4000 cm^−1^. The peaks located at 1731.7 and 1636.9 cm^−1^ are the characteristic peaks of carbonyl group stretching from aldehydes and ketones [16]. According to Figure 2, a broad band at 3420.6 cm^−1^ is due to –OH group stretching. Also two bands which can be seen at 1376.5 and 1512.7 cm^−1^ are related to COO– group on H_3_PO_4_-treated rice husk surface and the peaks at 2928.1 cm^−1^ represent the C–H stretching, respectively [27, 28]. It can be concluded that hydroxyl, carboxyl, and hydrocarbyl groups were the main components of H_3_PO_4_-treated rice husk. It also shows that after adsorbing Cu(II), the peaks at 3420.6 cm^−1^, 2928.1 cm^−1^, and 1095.8 cm^−1^ reduced to 3411.5 cm^−1^, 2921.5 cm^−1^, and 1091.5 cm^−1^, respectively. This fact indicated the surface functional groups of H_3_PO_4_-treated rice husk could combine with Cu(II) intensively. These groups had been reported to enhance metal ions adsorption [29].

### 3.2. Effect of Initial pH

**Figure 3:**
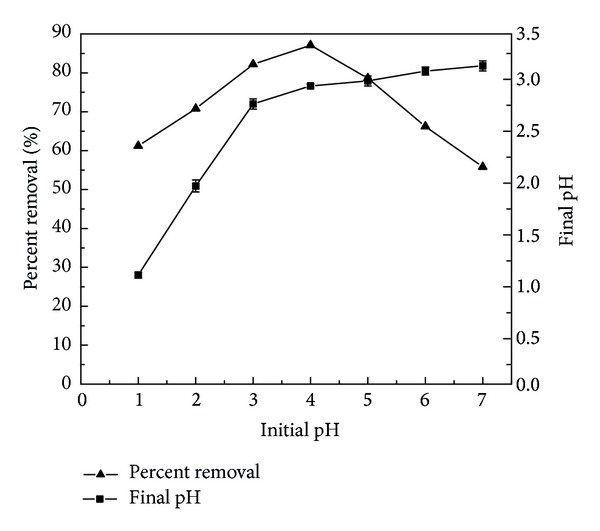
Effect of initial pH on the removal of Cu(II) (concentration of Cu(II) ions: 5 mg/L; mass of adsorbents: 2 g/L; temperature: 25°C).

Change in initial pH affects the adsorptive process through dissociation of functional groups on the active sites on the surface of the adsorbent [5]. The study on the effect of pH on the sorption of Cu(II) by H_3_PO_4_-treated rice husk would be very important in establishing the optimum of adsorptive process in the solutions and the results are shown in Figure 3.

The initial pH was ranged from 1 to 7, because Cu(II) would get converted to copper hydroxide and get precipitated above pH 8. So it could be concluded that the removal of Cu(II) was due to adsorption but not due to precipitation when the pH value was between 1 and 7. The results show that with the increasing of initial pH the removal efficiency of Cu(II) increases firstly but decreases after the pH reaches 4. The maximum removal was 87.1% at pH 4. This phenomenon is partly attributed to the fact that substantial hydrogen ions compete for vacant adsorption sites of adsorbent and protonation of active sites of H_3_PO_4_-treated rice husk thus leading to the extensive repulsion of copper ions at lower pH [30]. Hence, the hydrogen ions could be adsorbed more easily than copper ions depending on their high ion migration rate and high ion concentration in the solution. On the other hand, the solution pH influences the ionization of the functional groups onto the adsorbent surfaces. As silica is an important constituent of rice husk, the uptake of Cu(II) can occur by cation exchange reaction through the substitution of protons from silanol groups on the surface with the copper ions in the solution. Besides, the heavy metal ions may form a complex with inorganic ligands such as hydroxyl group. This fact can be confirmed further by the decrease of the final solution pH. It is more significant at higher pH.

### 3.3. Effect of Contact Time

**Figure 4:**
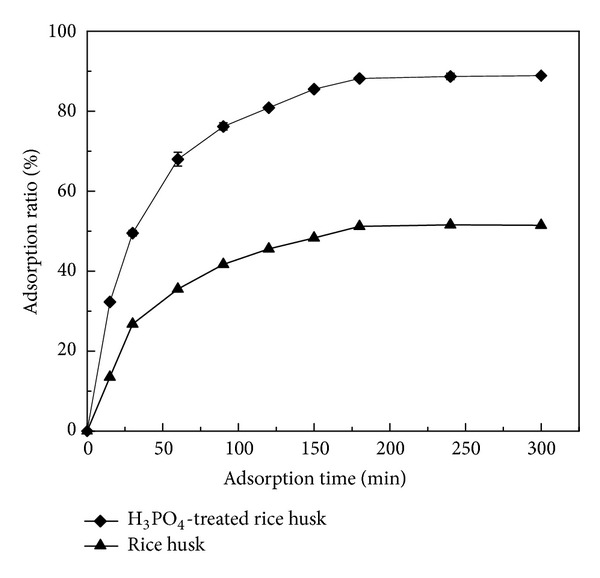
Effect of contact time on adsorption of Cu(II) by H_3_PO_4_-treated rice husk (pH: 4; concentration of Cu(II) ions: 5 mg/L; mass of adsorbents: 2 g/L; temperature: 25°C).

The effect of contact time on the uptake of Cu(II) ions onto H_3_PO_4_-treated rice husk and untreated rice husk was studied and is shown in Figure 4.

It can be observed that the rate of Cu(II) adsorption increased sharply in the first 90 min, but then the increase of adsorption rate tended to be gradual and eventually reached a plateau after 180 min. The maximum adsorption percentage was 88.9%. This phenomenon may be related to the vacant adsorption sites on the adsorbent surface. During the initial stage of sorption, a large number of vacant surface sites are available for adsorption. After lapse of time, the remaining vacant surface sites can be occupied difficultly due to repulsive forces between the solute molecules on the adsorbent surface and the bulk phase. This phenomenon has also been proven by Wongjunda and Saueprasearsit [31]. Since the adsorption ratio after 180 min was approximate to that after 300 min, the adsorption reaction time can therefore be retained at 180 min. Adsorption kinetics, which is one of the important characteristics defining the efficiency of sorption, described the solute uptake rate. The kinetics of Cu(II) adsorption was evaluated by applying two common models: (1) the pseudo-first-order kinetic model [32] and (2) the pseudo-second-order kinetic model [33].

The pseudo-first-order kinetic model assumes that the uptake rate of Cu(II) with time is directly proportional to the amount of available active sites on the adsorbent surface. The general equation is expressed as

(1) $$\begin{array}{c} \frac{{dq}_{t}}{dt}=K_{1}\left(q_{e}-q_{t}\right), \end{array}$$

where *q*_*e*_ and *q*_*t*_ are the uptake amount (mg/g) at equilibrium and at time *t*, respectively, and *K*_1_ is the pseudo-first-order adsorption rate constant (min^−1^). Integrating (1) for the boundary condition *t* = 0 to *t* = *t* and *q*_*t*_ = 0 to *q*_*t*_ = *q*_*t*_, the linear form of (1) becomes

(2) $$\begin{array}{c} \log\left(q_{e}-q_{t}\right)=\log q_{e}-\frac{K_{1}t}{2.303}. \end{array}$$

**Figure 5:**
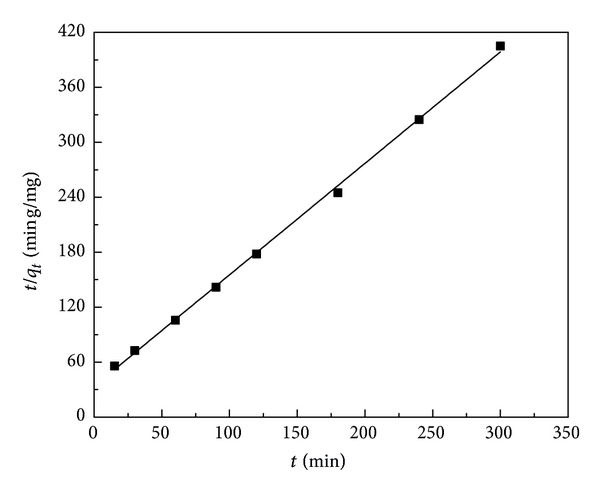
Pseudo-second-order kinetic plot for the Cu(II) adsorption by H_3_PO_4_-treated rice husk (pH: 4; concentration of Cu(II) ions: 5 mg/L; mass of adsorbents: 2 g/L; temperature: 25°C).

The values of adsorption rate constant were determined from the plot of log (*q*_*e*_ − *q*_*t*_) against *t* (not shown) and (2). The pseudo-second-order kinetic model assumes that chemical adsorption can be the rate limiting stage involving valence forces through sharing or exchange of electrons between adsorbent and adsorbate. The pseudo-second-order kinetic equation and the linear form are

(3) $$\begin{array}{c} \frac{{dq}_{t}}{dt}\;=K_{2}{\left(q_{e}-q_{t}\right)}^{2},\\ \frac{t}{q_{t}}=\frac{1}{K_{2}{q_{e}}^{2}}+\frac{t}{q_{e}}, \end{array}$$

where *K*_2_ is the pseudo second-order adsorption rate constant (g/mg·min). The initial uptake rate can be obtained as *q*_*e*_/*t* approaches zero:

(4) $$\begin{array}{c} h_{0}=K_{2}{q_{e}}^{2}, \end{array}$$

where *h*_0_ is the initial adsorption rate (mg/g·min). The rate constant and correlation coefficient can be calculated based on the plot (Figure 5) of *t*/*q*_*t*_ versus t for Cu(II) adsorption.

The rate constants and correlation coefficients of the two kinetic models for the Cu(II) adsorption are presented in Table 3.

Results show that the pseudo-second-order model was more appropriate for the adsorption of Cu(II). The correlation coefficients of adsorption using rice husk and H_3_PO_4_-treated rice husk for pseudo-second-order kinetic model are both closer to unity than those for the pseudo-first-order kinetic model. And the theoretical *q*_*e*_ values of the H_3_PO_4_-treated rice husk agree with the experimental *q*_*e*_ values data better than those for the pseudo-first-order kinetic model. While the theoretical *q*_*e*_ values of the rice husk adsorption for the two kinetic models both agree with the experimental *q*_*e*_ values well, the kinetic models fit well with the adsorption process and confirm the chemisorption of Cu(II) onto rice husk.

### 3.4. Study of Adsorption Isotherms

**Figure 6:**
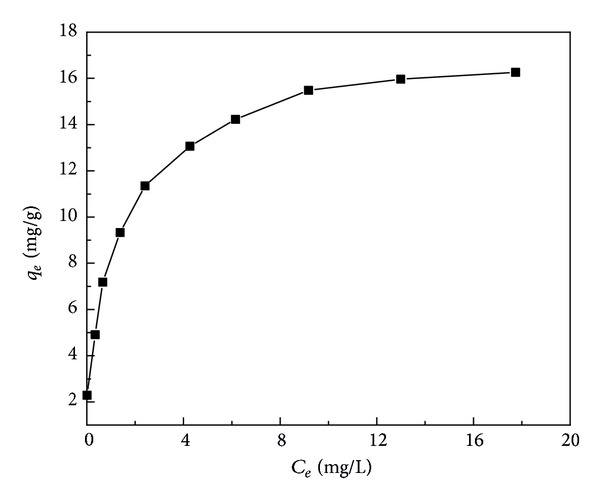
Adsorption isotherms for Cu(II) by H_3_PO_4_-treated rice husk (mass of adsorbents: 2 g/L; pH: 4; temperature: 25°C).

Adsorption isotherms described the adsorption process and the interaction between adsorbates and adsorbents. It is important to establish the most acceptable correlations for the batch equilibrium data for analysis and design of adsorption systems. The Langmuir and Freundlich models are the most frequently used to describe the equilibrium data of adsorption. In the present work, these two models were applied on the study of adsorption isotherms of Cu(II).  Figure 6 depicts the adsorption equilibrium experimental data of Cu(II) onto H_3_PO_4_-treated rice husk.

The Langmuir model assumes that the uptake of metal ions is on a homogenous surface and all the adsorption sites are energetically identical without any interaction between adsorbed ions [34]. The expressions of Langmuir equation and the linear form are

(5) $$\begin{array}{c} q_{e}=\frac{q_{m}k_{a}C_{e}}{\left(1+k_{a}C_{e}\right)},\\ \frac{C_{e}}{q_{e}}=\frac{1}{q_{m}k_{a}}+\frac{C_{e}}{q_{m}}, \end{array}$$

where *C*_*e*_ is the equilibrium concentration of Cu(II) in solutions (mg/L), *q*_*m*_ is the maximum uptake amount per g of adsorbent (mg/g), and *k*_*a*_ is the Langmuir constant related to binding energy of the sorption system (L/mg). The Langmuir parameters, *q*_*m*_ and *k*_*a*_, were calculated from the linear plots of *c*_*e*_/*q*_*e*_ against *c*_*e*_ (not shown). The values of parameters were calculated and are shown in Table 4.

Within the Langmuir model, the saturation adsorption capacity *q*_*m*_ is supposed to coincide with saturation of a fixed number of identical surface sites and as such, it should logically be independent of temperature [35]. Compared to the Freundlich isotherm, the Langmuir isotherm model fitted better with the sorption process. The chemical modification of rice husk resulted in the increase of its adsorption capability. The equilibrium sorption data can fit well with the Langmuir model based on the value of correlation coefficient *R* ^2^.

The Freundlich model assumes a heterogeneous surface with a nonuniform distribution of heat of adsorption over the surface, and the adsorption sites are distributed exponentially with respect to the heat of adsorption [36]. The Freundlich isotherm can be described as follows:

(6) $$\begin{array}{c} q_{e}=K_{F}{C_{e}}^{1/n}. \end{array}$$

The linear form of the Freundlich isotherm is given by

(7) $$\begin{array}{c} \log q_{e}=\log K_{F}+\left(\frac{1}{n}\right)\log C_{e}, \end{array}$$

where *K*_*F*_ is the Freundlich constant indicative of the relative adsorption capacity of the adsorbent and the constant 1/*n* indicates the adsorption intensity. Smaller value of 1/*n* implies stronger interaction between the adsorbent and heavy metal while 1/*n* equal to 1 indicates linear adsorption leading to identical adsorption energies for all sites [37]. The parameters of Freundlich isotherm are shown in Table 4. Among the limited concentrations, the fact that the experimental data fitted well with the Freundlich isotherm model indicates that the surface of the adsorbent was nonuniform.

## 4. Conclusions

H_3_PO_4_-treated rice husk could be better used as an adsorbent material for the removal of copper(II). The maximum sorption amount for Cu(II) was 17.0358 mg/g at pH 4. The adsorption process reached equilibrium within 180 min. The SEM image of H_3_PO_4_-treated rice husk showed that the porous structure of the sorbent surface could improve the adsorption capacity. The FT-IR technology identified the presence of acidic functional groups, such as carbonyl, carboxyl, and hydroxyl. It is significative to consider the biological adsorbent regeneration treatment, the analysis of operational cost and economic cost, then provides a reference for practical engineering application in the future.

## Figures and Tables

**Table 1:** Main experimental instruments and analysis items.

Determination item	Method	Instrument
Determination of Cu(II) concentrations	Atomic absorption spectrometry	Atomic absorption spectrophotometer (Model AA6800, Shimadzu, Japan)

Element composition of raw rice husk and H_3_PO_4_-treated rice husk	X-ray fluorescence (XRF) spectrum	X-ray fluorescence spectrometer (Model Axios PW4400, PANalytical)

Analysis of functional groups may be present	FT-IR spectrum	Fourier Transform Infrared spectrophotometer (Nicolet Magne 750)

Surface morphology	Scanning electron microscopic (SEM) analysis	Scanning electron microscope (Model S-3400N, HITACHI)

**Table 2:** Composition of rice husk by XRF analysis.

Element component	Native rice husk (wt.%)	H_3_PO_4_-treated rice husk (wt.%)
O	25.982	24.819
Na	0.019	0.025
Mg	0.06	0.018
Al	0.038	0.040
Si	15.367	17.927
P	0.065	0.349
S	0.130	0.098
Cl	0.234	0.050
K	1.209	—
Ca	0.399	0.148
Mn	0.039	0.018
Fe	0.014	0.026

**Table 3:** Kinetic parameters for the adsorption of Cu(II) by rice husk and H_3_PO_4_-treated rice husk.

Adsorbent	*q*_*e*,exp⁡_ (mg/g)	Pseudo-first-order model			Pseudo-second-order model			
		*K*_1_ (min^−1^)	*q*_*e*_ (mg/g)	*R* ^2^	*K*_2_ (g/mg·min)	*q*_*e*_ (mg/g)	*h*_*o*_ (mg/g·min)	*R* ^2^
Rice husk	0.4300	0.0219	0.4759	0.9507	0.0588	0.4888	0.0140	0.9974
H_3_PO_4_-treated rice husk	0.7408	0.0253	0.8498	0.9730	0.0440	0.8218	0.0300	0.9988

**Table 4:** Isotherm parameters for the adsorption of Cu(II) by rice husk and H_3_PO_4_-treated rice husk.

Adsorbent	Freundlich model			Langmuir model		
	*K*_*F*_	1/n	R^2^	K_a_ (L/mg)	q_m_ (mg/g)	R^2^
Rice husk	0.4267	0.4197	0.9402	0.3403	1.6173	0.9559
H_3_PO_4_-treated rice husk	7.1187	0.3594	0.9303	0.9915	17.0358	0.999
